# The Value of Long-Term Stream Invertebrate Data Collected by Citizen Scientists

**DOI:** 10.1371/journal.pone.0153713

**Published:** 2016-04-27

**Authors:** Patrick M. Edwards

**Affiliations:** Portland State University, Environmental Sciences and Management, Portland, Oregon, United States of America; University of Bologna, ITALY

## Abstract

The purpose of this investigation was to systematically examine the variability associated with temporally-oriented invertebrate data collected by citizen scientists and consider the value of such data for use in stream management. Variability in invertebrate data was estimated for three sources of variation: sampling, within-reach spatial and long-term temporal. Long-term temporal data were also evaluated using ordinations and an Index of Biotic Integrity (IBI). Through two separate investigations over an 11-year study period, participants collected more than 400 within-reach samples during 44 sampling events at three streams in the western United States. Within-reach invertebrate abundance coefficient of variation (CV) ranged from 0.44–0.50 with approximately 62% of the observed variation strictly due to sampling. Long-term temporal CV ranged from 0.31–0.36 with 27–30% of the observed variation in invertebrate abundance related to climate conditions (El Niño strength) and sampling year. Ordinations showed that citizen-generated assemblage data could reliably detect differences between study streams and seasons. IBI scores were significantly different between streams but not seasons. The findings of this study suggest that citizen data would likely detect a change in mean invertebrate density greater than 50% and would also be useful for monitoring changes in assemblage. The information presented here will help stream managers interpret and evaluate changes to the stream invertebrate community detected by citizen-based programs.

## Introduction

Public interest in environmental issues provides an opportunity to engage citizens in collecting data for natural resource management [1–6]. This is particularly true of natural surface water systems, where a growing number of governmental and private organizations encourage and support the use of citizen-based data to monitor streams and watershed health [7]. Many of these organizations use stream macroinvertebrate (invertebrate) bioassessment as a tool for stream monitoring and evaluation [7–10]. Stream invertebrates are known to be reliable indicators of stream habitat integrity [11, 12] and are commonly used in stream management [13, 14]; therefore, many citizen-based programs are focused on collecting invertebrates as an approach to stream monitoring as well as a strategy for enhancing science education and promoting environmental stewardship.

While the value of long-term ecological data is well known [15], there are relatively few studies of stream invertebrates with durations longer than five years [16]. The lack of long-term invertebrate data represents an area in which citizen scientists could make valuable contributions to stream management. This has been observed in other scientific disciplines. For example, a recent study in the field of ornithology found that long-term data collected by citizen groups was cited in 24–77% of the published research about the impact of climate change on avian migration [17]. Routine monitoring of the same stream site is logistically convenient for citizen-based programs and would serve the dual purpose of engaging the public in environmental issues and generate valuable long-term data sets. However, there is concern about the quality of stream invertebrate data collected by non-professionals and its use in stream management [18]. These concerns are primarily focused on aspects of invertebrate sampling that present unique challenges to citizen groups and thereby increase data variability, bias and uncertainty.

**Fig 1:**
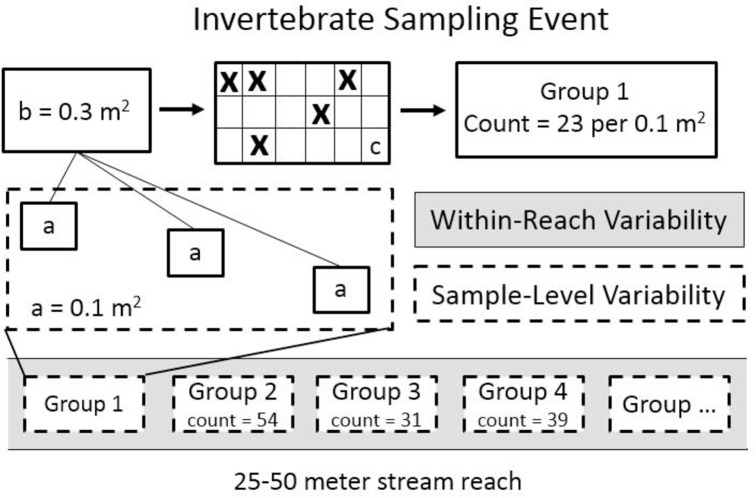
Illustrates a typical sampling event using the Field method and presents two sources of variability related to citizen-generated invertebrate data. In this example, four groups collected a total of 147 invertebrates (mean density = 36.8 per m^2^) from a 100 m stream reach. Participants collected three 0.1m^2^ D-net benthic samples from the left, center and right of a stream riffle (a). The invertebrates were composited into a plastic tub (b), transferred to the subsampling tray (c) and invertebrates from five randomly-selected cells in the tray (X) were sorted and counted. Sample-level variability is related to the process of subsampling. Reach-level variability is due to both sample-level variation and reach-scale spatial differences in the invertebrate community. Variability is hierarchically structured with sample-level variability nested within the reach-level samples.

There are three main sources of variability inherent to long-term stream invertebrate data generated by citizen groups: sampling variation, within-reach spatial variation and site-level temporal variation. Sampling variation (sample-level) occurs when invertebrates from a benthic sample are sorted from debris and counted in the field [4]. In a typical invertebrate sample, there are far too many organisms to sort and count in a reasonable amount of time. Through a process called subsampling, a randomized subset of invertebrates is selected and used to represent the entire sample population [19]. In professional science, subsampling is conducted in the laboratory using artificial lighting and magnification (10x). However, because many citizen-based programs prefer to return invertebrates to the stream alive, subsampling takes place in the field and therefore presents a major challenge to ensuring data quality [20–24]. The second source of variability (within-reach) is spatially-oriented and is due to repeated sampling of invertebrates in different locations within the study reach. Within-reach variability has two main components: variability due to the process of collecting and subsampling (sample-level) and the small-scale spatial differences of the invertebrate community within the stream (Fig 1). The third component of variability is long-term temporal variation at the site-level and is due to changes in invertebrate abundance over time (temporal). Temporal variation is composed of environmental, interannual and within-reach sources of variability.

While several published studies have examined the use of citizen-generated data to assess stream condition across a spatial disturbance gradient [3,23], there are no studies of long-term stream invertebrate data collected by citizen scientists or other non-professionals. Furthermore, there are no studies that attempt to systematically characterize and estimate the variability in stream invertebrate data collected by citizen groups. Such information would be valuable to both stream managers and citizen-based stream monitoring programs. The goal of this study was to estimate and evaluate three components of variability inherent to long-term invertebrate data typically collected by citizen-based stream monitoring programs. This was accomplished by: 1) characterizing and estimating three sources of variability related to invertebrate data generated by citizen scientists, and 2) evaluating the use of data generated by citizen science programs to detect changes in the stream invertebrate community.

## Methods

### 2.1 Participants and study sites

**Fig 2:**
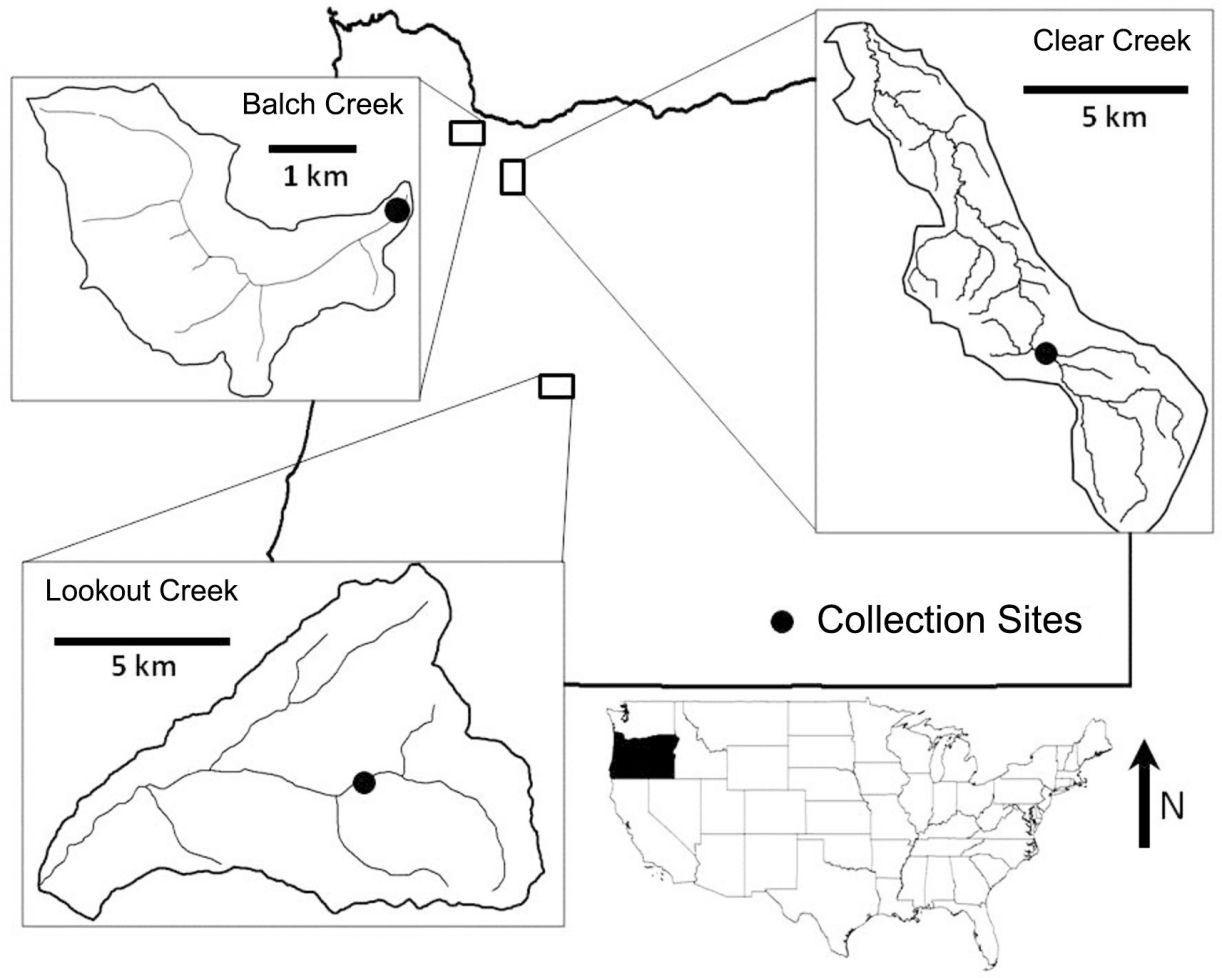
Map of study watersheds and data collection sites in Oregon, United States. Black circles show data collection sites. Map scale is indicated within each watershed map.

This study was accomplished through two investigations that were conducted at three streams in western Oregon, United States (Fig 2). In both investigations, college students collected invertebrates using the nonlethal field-based sampling technique described below in section 2.2. In 2004, the Portland State University Office of Research Integrity reviewed and approved the proposed research. Participants were verbally informed that the data would be used for research and made publicly available. The majority of participants were non-science majors and did not receive any training prior to the first field collection. For this reason, the student’s abilities were considered to be similar to those of a typical citizen scientist or other non-professional. All training, sampling and identification took place during two-hour field trips. The first investigation, which was conducted in the spring and summer of 2007 at Balch Creek and Clear Creek, examined the variability associated with a field-based invertebrate subsampling method. In the second investigation, 44 sampling events from 2005–2015 were used to estimate and evaluate both within-reach and long-term temporal variation at Balch and Lookout Creeks. All invertebrates were identified to family level and included taxa from five major insect orders (mayfly, stonefly, caddisfly, true fly (Diptera) and beetle) and other non-insect taxa including Gammaridae, Gastropods and Oligochaetes. Mites were excluded from analysis because their small size makes them difficult to see in the field and count.

Study streams were located in the maritime climate of western Oregon and situated in relatively steep forested watersheds. Vegetation cover in all three watersheds was primarily Douglas fir (*Pseudotsuga menziesii)* and Western hemlock (*Tsuga heterophylla*). Two criteria were used to select three stream sites in three separate watersheds with varying levels of land development. First, the stream reach had to be conveniently located and safely accessible for large groups of participants. Second, the invertebrate communities had to be different at each site, which was determined through prior exploratory sampling. All three study streams were generally small (less than 10 meters wide) and shallow (less than 0.5 meters deep) with clear cold stream water flowing rapidly over rocky substrates. The Balch Creek watershed has an area of 6 km^2^ and the sampling site was located at 15 m elevation (above mean sea level). The majority of the Balch Creek watershed is situated within a forested city park while the remainder (approximately 20%) is moderately developed. The Clear Creek watershed is undeveloped and located in the western foothills of the Cascades mountain range. The collection site on Clear Creek was located approximately midpoint in the basin with a contributing area of 73 km^2^ and an elevation of 62 m. Lookout Creek is also undeveloped and is located in the western Cascades at the HJ Andrews Experimental Forest. The Lookout Creek collection site is located at an elevation of 490 m in an old growth forest situated in the upper part of the watershed and with a contributing basin area of 16 km^2^. Field permits were obtained from Portland Parks and Recreation and the US Forest Service, HJ Andrews Experimental Forest. Given the differences in ecological and environmental characteristics of each watershed, it was expected that citizen data would consistently and reliably detect the different invertebrate communities in each stream system.

### 2.2 Nonlethal Field method for citizen science

For all investigations, participants used the same nonlethal field-based technique to collect and subsample invertebrates. A description of the method follows (from here forward referred to as the Field method). Groups with 3–5 participants used D-nets to collect three benthic samples in riffles from the left, center and right of the stream (Fig 1). Participants were directed to collect for about one minute and sample 1 ft^2^ (0.1 m^2^) of substrate area in front of the net. To avoid injuring or killing invertebrates, participants collected samples by hand-disturbing the substrate and gently rubbing the surface of large rocks. The technique was demonstrated in-stream to the participants. The three D-net samples were composited into a plastic tub so that each group’s sample represented approximately 0.3 m^2^ of the stream bottom sampled. Subsampling was accomplished using a plastic storage container as a sorting tray (Akro Mils, 38 cm X 24 cm x 6 cm, part #05905). The bottom half of the container, which is divided into 18 cells (8 cm x 6 cm x 6 cm), was used to hold the insects as they were subsampled. The bottoms of the cells were sealed with silicone to prevent invertebrate from moving between partitions. The 0.3 m^2^ composite sample was poured from the tub into the sorting tray where invertebrates were picked from five randomly selected cells representing approximately one-third of the tray area (not all cells are the same size) and approximately 0.1 m^2^ of stream substrate area. Next, participants sorted invertebrates from debris with plastic pipettes and no-crush forceps and transferred invertebrates to ice-cube trays filled with clear water where they sorted, identified and counted invertebrates. Participants used magnifying glasses and ambient light conditions to sort and pick invertebrate samples. Finally, an experienced taxonomist verified the counts, identifications and data sheets. After verification, the invertebrates were returned to the stream alive. Each group of participants followed this procedure, thereby generating multiple within-reach samples that were combined into one sampling event. For each reach, the data from each group were summed and expressed as total abundance per 0.1 m^2^ (Fig 1).

### 2.3 Sample-level variability

In the first investigation, the variability due to subsampling (sample-level) was estimated and evaluated for the Field method. This was accomplished by quantifying the variation of the Field method and comparing it to a laboratory-based method for sorting and subsampling. Invertebrate samples were obtained by participants using the Field method to collect six samples at both Balch and Clear Creeks (Table 1). An experienced taxonomist verified the counts in the field and then preserved the subsamples and remaining samples separately in 70% ethanol. In the laboratory, the subsample was returned to the remaining sample matrix and then re-subsampled using a Caton tray, artificial lighting and magnification (10x). The Caton tray is a metal subsampling tray with 24 cells and a 370-micron mesh bottom [25]. Invertebrate samples were poured into the Caton tray and sorted from randomly selected cells until a count similar to the Field subsample was achieved (Lab method). The remainder of the sample was also counted and added to the Lab subsample count to determine the total invertebrate count of each sample (Total). The Total count was used to develop a statistical Null-hypothesis model (Null), which was a statistical representation of the potential variation due to subsampling. The Null model was developed using an R loop to repeatedly re-subsample (N = 1000) the total count for each sample from both streams (n = 6 at each stream). For example, if the Field and Lab subsamples generated a count of 183 and 179 respectively, the Total count was randomly subsampled to the median value (181) 1,000 times. The mean relative abundance of the Null model was used as a statistical benchmark to determine relative abundance variation for each taxon as well as to compare assemblage data generated by both the Field and Lab methods. For each taxon collected at each stream, the deviation in relative abundance (Y Taxon Deviation) was determined by subtracting the observed relative abundance value for each method (X Observed) from the Null model mean relative abundance value (X Null) and standardizing the percent difference to the Null model mean (Eq 1). The mean taxon deviation for both streams (n = 6 at each stream) was used to estimate variation due to subsampling.

YTaxonDeviation=(XObserved−XNull)/XNull (1)

For both the Field and Lab methods, mean deviation values per sample were normally distributed and statistically evaluated using a two-tailed paired t-test.

**Table 1:** Summary data from the investigation comparing the Field and Lab methods.

*Method and Location*	*n*	*Total Abundance*	*Mean (SD)*	*Min-Max*
Field Clear Creek	6	893	148 (72)	71–265
Lab Clear Creek	6	907	151 (80)	72–287
Field Balch Creek	6	691	115 (69)	42–233
Lab Balch Creek	6	719	120 (72)	43–239

The effect of sample-level variability on invertebrate assemblage was evaluated using ordinations. Ordinations are often used to display biological community data in a way that allows for visual and statistical evaluation. Ordinations of invertebrate relative abundance were generated using Non-metric Multidimensional Scaling (NMDS) based on Bray-Curtis distance. NMDS is an unconstrained technique that examines the overall similarity of a biologic community among samples in an ordination. NMDS is often used with invertebrate data because it preserves the inter-site rank relationships and thus better represents species distance [26, 27]. Two-dimensional ordinations were generated for the Field, Lab and Null Model assemblage data and evaluated using the statistical tests known as Analysis of Similarities (ANOSIM) and Procrustes [28, 29]. ANOSIM statistically compares ordinations and was used to test the hypothesis that there is no difference between invertebrate assemblage between streams [27]. The ANOSIM test statistic (R) ranges from 0–1. An R value of 0 indicates random grouping between invertebrate samples and a value of 1 indicates a 100% dissimilarity between invertebrate samples. The statistical significance of R is calculated with a permutation test (N = 1,000). Procrustes analysis was used to compare the paired Field and Lab samples at both streams. Procrustes maximizes the similarities between ordinations and displays them in a way that allows for sample-to-sample comparison [26, 27]. The Procrustes test statistic (M^2^) is a measure of similarity and the permutation-based Protest is used to determine the statistical significant of M^2^ [28]. The M^2^ statistic ranges from 0–1 and is a measure of correspondence between the rotated matrices. M^2^ values close to 1 indicate concordance between paired samples in an ordination [29]. Procrustes and Protest were used to determine if the ordinations generated by the Field (n = 6) and Lab (n = 6) methods were statistically similar at each creek (N = 1,000 permutations). All statistical analyses for both investigations were performed with R statistical software using the default settings for both the “vegan” and “relaimpo” packages [30, 31].

### 2.4 Within-reach spatial variability

In the second investigation, within-reach spatial variability was estimated by calculating the mean Coefficient of Variation (CV) of invertebrate abundance of group-level samples and was evaluated using ordinations and an Index of Biotic Integrity (IBI). Data for this investigation were collected at Balch Creek and Lookout Creek each spring (n = 11) and fall (n = 11) from 2005–2015. At each stream, 5–14 groups of participants sampled the same reach collecting a total of 248 and 155 within-reach samples at Balch Creek and Lookout Creek respectively (Table 2). For each sampling event, invertebrate density CV of the group-level samples was calculated for Balch Creek (n = 22 sampling events) and for Lookout Creek (n = 21 sampling events). For one sampling event (fall 2005) at Lookout Creek, only the total invertebrate count was recorded, so reach-level CV could not be calculated. Within-reach CV was partitioned using the mean percent taxon deviation (62%) due to subsampling determined in the first investigation. Within-reach CV was normally distributed and differences between streams and seasons were evaluated using a two-tailed t-test.

**Table 2:** Summary of long-term invertebrate data collected by participants from 2005–2015. Annual sampling did not occur on the same day each year, so the range of sampling days over the 11-year study period is shown as fall and spring Sampling dates. Total area sampled is the combined area sampled by all groups during each sampling event. Sample event abundance is the total number of invertebrates collected during each sampling event. Mean density is expressed as number of invertebrates per 0.1 m^2^.

*Creek*	*Within -Reach Samples*	*Total Sample Events*	*Fall Sampling Dates*	*Spring Sampling Dates*	*Total Area Sampled (m* ^*2*^ *)*	*Sample Event Abundance*	*Mean Density (per 0*.*1 m* ^*2*^ *)*
Balch	248	22	Oct 30-Nov 10	May 3–13	0.5–2.5	103–811	29
Lookout	155	22	Nov 9–22	May 9–26	0.5–1.0	106–529	39

Assemblage data generated for each sampling event (n = 22 at each creek) were evaluated using ordinations and a family-level IBI [32]. Ordinations were used to examine patterns in assemblage data over the 11-year study period. Ordinations were generated using relative abundance data derived from the total invertebrate abundance collected during each sampling event. Ordinations were statistically evaluated using ANOSIM to determine if invertebrate assemblage was significantly different between streams (n = 22 at each stream) and seasons (n = 11 for each season at each stream). IBIs are a metric commonly used by watershed managers to characterize stream condition. The IBI used in this analysis is comprised of six metrics: richness (total, mayfly, stonefly caddisfly), % Diptera and % dominance. Each metric is scored as a 1, 3 or 5 and the total score is summed (range 6–30) to categorize stream condition as either severely impaired (< 16), moderately impaired (17–23) or unimpaired (> 23). IBI scores were determined using the total counts from each sampling event over the 11-year study period (Fig 1). IBI scores were not normally distributed and were statistically evaluated for each stream and season using a rank-based test (Mann Whitney test).

### 2.5 Site-level temporal variability

For all sampling events over the 11-year study period (n = 22 at each stream), site-level temporal variation was estimated by calculating CV for total invertebrate abundance and 20 invertebrate families. Temporal variation in invertebrate abundance was evaluated using linear regression and a permutation-based approach to decompose the multiple-linear model R^2^ into relative contributions for each predictor term [33]. To estimate the climactic component in temporal variation, the Multivariate El Niño Southern Oscillation Index (ENSO MEI) was used as a broad-scale proxy for climate condition. The ENSO MEI index is a monthly measure of ENSO signal strength derived from six ocean-atmosphere variables [34]. The ENSO MEI is a scaled value with negative values indicating La Niña conditions and positive values indicating El Niño conditions. In this analysis, ENSO MEI values ranged from -2.00 to 2.5. ENSO strength is known to be associated with several aspects of the stream invertebrate community including abundance [35], richness [35, 36] and persistence [37]. ENSO MEI values were obtained from NOAA Earth Centers Research Laboratory (accessed online November 2015: http://www.esrl.noaa.gov/psd/enso/mei/table.html). For each sample, the mean monthly ENSO MEI value was determined for the water-year prior to invertebrate sampling. Water-year dates were September-October for fall samples and September-April for spring samples. To evaluate the environmental component of temporal variation, a two-parameter linear regression model was developed using mean invertebrate abundance (log transformed) as a function of climate (ENSO MEI) and sample-year. The purpose for using sample-year in the multiple-linear model was to capture temporal variance that may be due to environmental conditions unrelated to climate. The final regression model used to partition temporal CV is described in Eq 2 below.

Ylog(meaninvertebratedensity)=B0+B1XENSOMEI+B2Xsample−year+Ɛ (2)

To determine the relative contribution of each predictor term in the multiple-linear regression model, the metric known as “LMG” (Relaimpo package, default settings) was used to decompose the overall linear model R^2^ into climactic and temporal components. LMG allows for the decomposition of the model R^2^ for each predictor term without the bias that occurs from the order of parameters in the model [33, 38]. Using the percent relative contributions from the LMG model, the observed long-term temporal CV was partitioned into either environmental (ENSO MEI + sample-year) or within-reach sources of variation.

## Results

### 3.1 Sample-level variability

**Fig 3:**
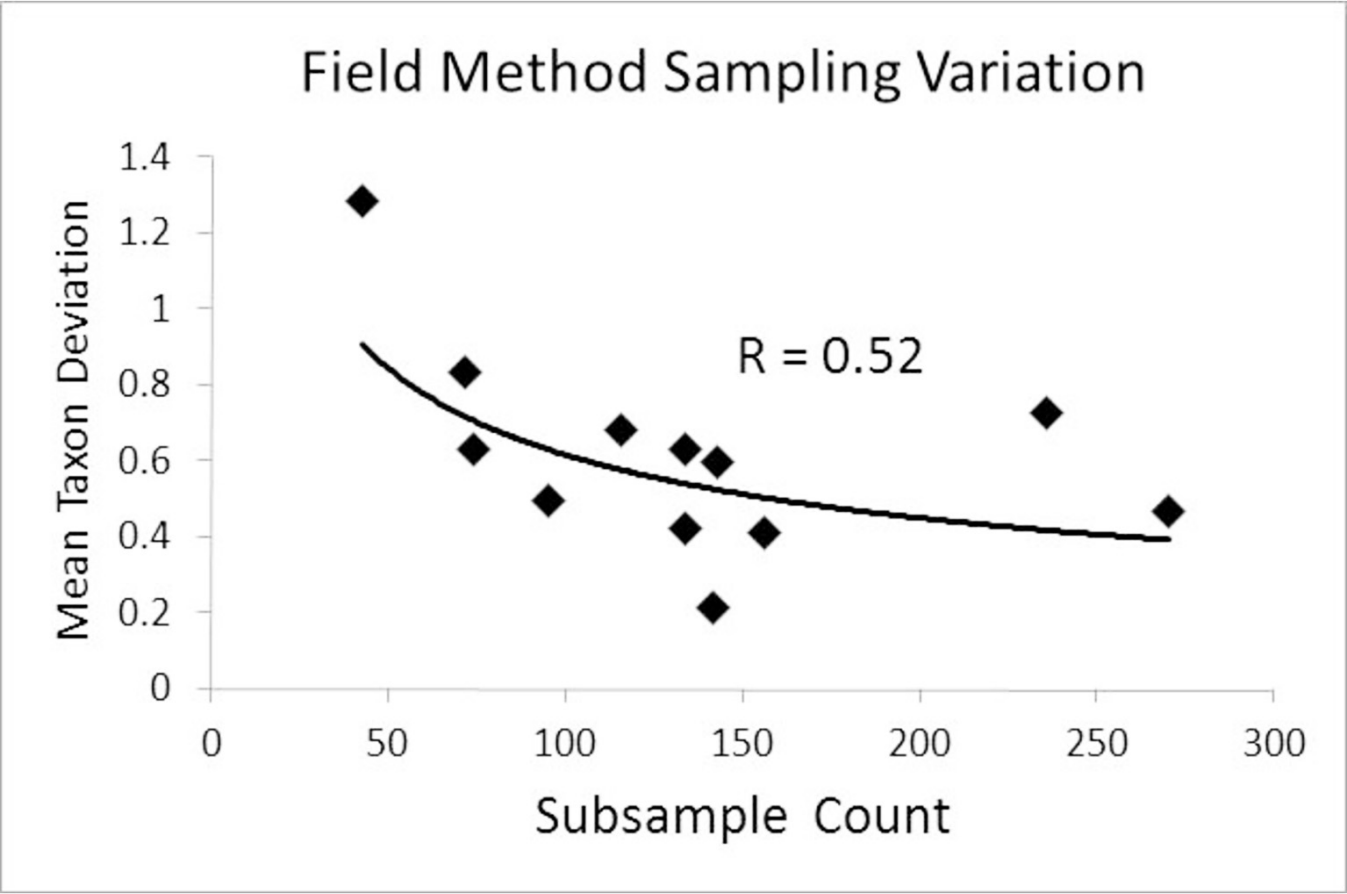
Mean taxon deviation of the Field method as a function of subsample count.

Using the Field method, six groups of participants subsampled approximately 700 invertebrates at Balch Creek and 900 at Clear Creek (Table 1). Mean Field subsample counts per group were 115 (range = 42–233) at Balch Creek and 148 (range = 71–265) at Clear Creek. Taxa richness ranged from 5–10 at Balch Creek and 8–13 at Clear Creek. The Lab method resulted in higher richness at both Clear Creek (mode = 1 taxon) and Balch Creek (mode = 3 taxa), mostly due to small invertebrates such as stoneflies (Chloroperlidae and Nemouridae) and beetles (Elmidae) that were missed in the Field samples. For taxa with mean deviations greater than 10% from the Null model mean, the Field method underestimated relative abundance in 29% of the taxa and overestimated it in 27% of the taxa. In the Lab samples, the underestimate and overestimate was 19% and 22% respectively. Underestimate bias in the Field method was associated with small taxa, while larger taxa (e.g. Rhyacophilidae, Heptageniidae) tended to be overestimated. For all taxa at each creek, the mean taxon deviation from the Null model mean was 62% (SD = 70%) for the Field method and 33% (SD = 32%) for the Lab method (t-Test, t = 3.3, *P* < 0.01, d.f. = 10). For taxa with greater than 5% relative abundance, mean taxon deviation dropped to 57% (SD = 23%) for the Field method and 32% (SD = 18%) for the Lab method (t-Test, t = 2.9, *P* < 0.01, d.f. = 10). Mean taxon deviation was negatively correlated with subsample count (R = 0.52) and appeared to stabilize at invertebrate counts between 100 and 150 (Fig 3).

**Fig 4:**
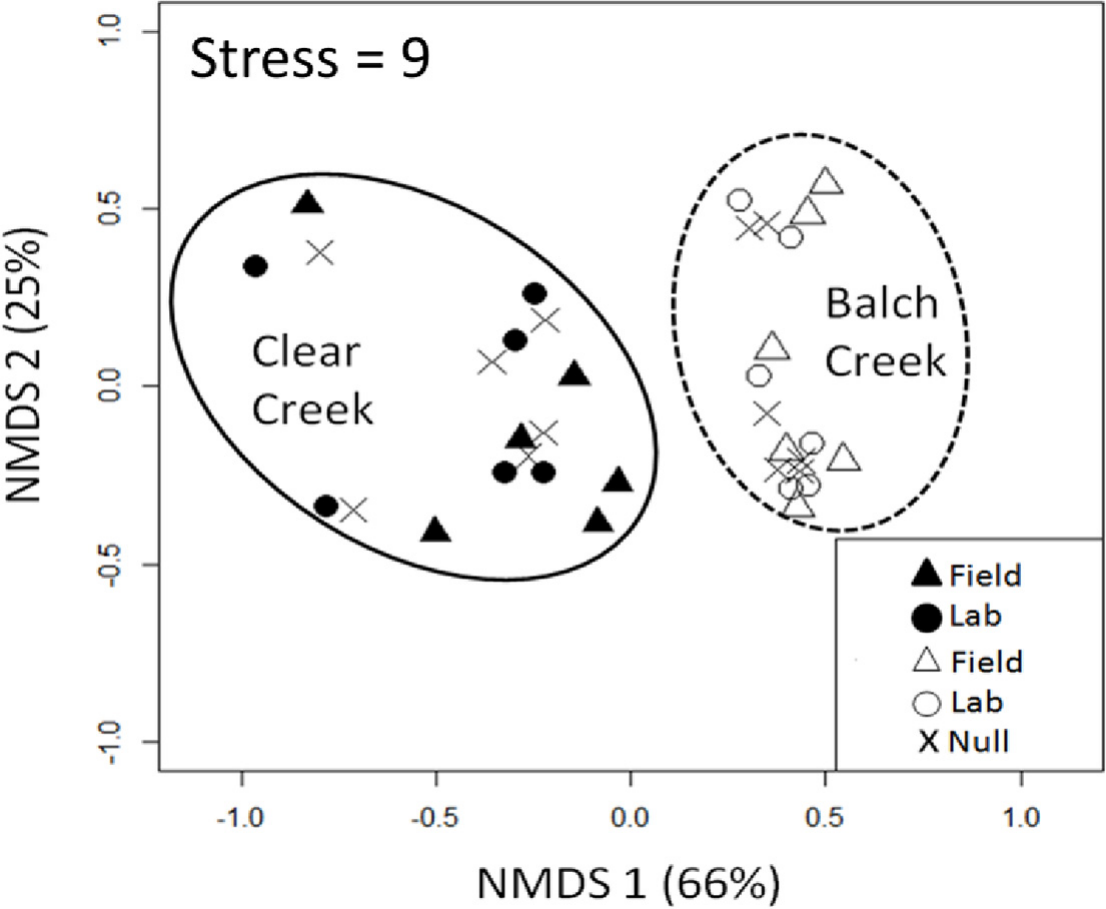
Ordinations of invertebrate assemblage at both Balch Creek (n = 6) and Clear Creeks (n = 6). Ordinations for the Field method (triangles, n = 6) and Lab method (circles, n = 6) are shown with the corresponding Null model assemblage (X, n = 6 at each creek). Symbols represent an individual sample collected at Clear Creek (closed symbols) and at Balch Creek (open symbols). Variance explained by each axis shown in the axis label.

Ordinations of relative abundance showed distinct clusters at Balch Creek and Clear Creek, indicating that the streams have different invertebrate assemblages (Fig 4). ANOSIM results showed that assemblages between streams were significantly different for both the Field (ANOSIM, R = 0.58, *P* < 0.01, n = 6 at each stream) and Lab Methods (ANOSIM, R = 0.71, *P* < 0.01, n = 6 at each stream). Invertebrate samples at each creek were separated along NMDS axis 1 and varied along NMDS axis 2. The assemblage at Clear Creek was more variable than Balch Creek, but in general the ordinations for both the Field and Lab methods were similar to the corresponding Null model pattern. The Procrustes analysis showed that both the Field and Lab methods generated similar ordinations at each stream (Protest, M^2^ = 0.94, *P* < 0.001, n = 6 at each stream). The length of the vectors in the Procrustes ordination indicated that sample-to-sample variance was small compared to the separation of the streams and the lack of pattern in the vectors suggests that the ordinations generated by the Field method were generally unbiased (S1 Fig).

### 3.2 Within-reach spatial variability

**Fig 5:**
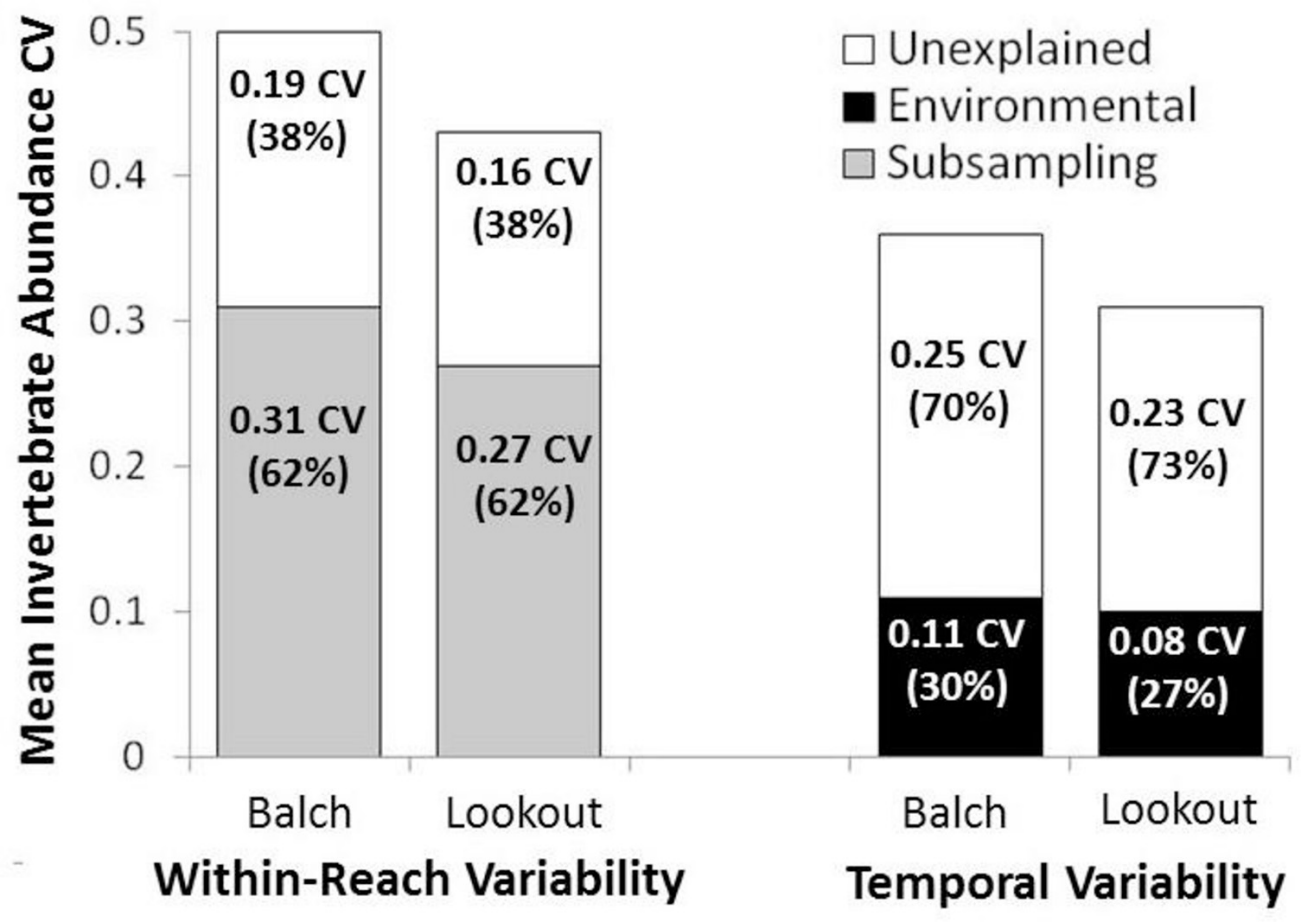
Partitioned invertebrate density CV for both within-reach spatial and long-term temporal citizen-generated data at Balch and Lookout Creeks. Within-reach spatial CV was partitioned into subsampling and unexplained components. Long-term temporal CV was partitioned into environmental (ENSO MEI + Sample-Year) and unexplained components.

From fall 2005 to fall 2015, approximately 700 participants from 25 classes collected, subsampled and identified invertebrates at Balch Creek and Lookout Creek (Table 2). Over the 11-year study period, 248 within-reach samples were collected at Balch Creek and 155 were collected at Lookout Creek. Each group of participants collected between 0 and 172 invertebrates per group-level sample. Total invertebrate abundance for each sampling event ranged from 103–811 (mean = 328) at Balch Creek and 106–529 (mean = 284) at Lookout Creek. Mean invertebrate abundance CV across all sampling events was 0.50 (range = 0.29–0.79) at Balch Creek and 0.44 (range = 0.24–0.70) at Lookout Creek. At both streams, mean CV was slightly higher in the spring but not significantly different from fall samples (t-Test, t = 1.2, *P* > 0.05, d.f. = 41). Using 62% as the mean deviation due to subsampling, the within-reach CV spatial variation due strictly to subsampling was estimated to be 0.31 at Balch Creek and 0.27 at Lookout Creek with the remainder of the variation unexplained (Fig 5).

**Fig 6:**
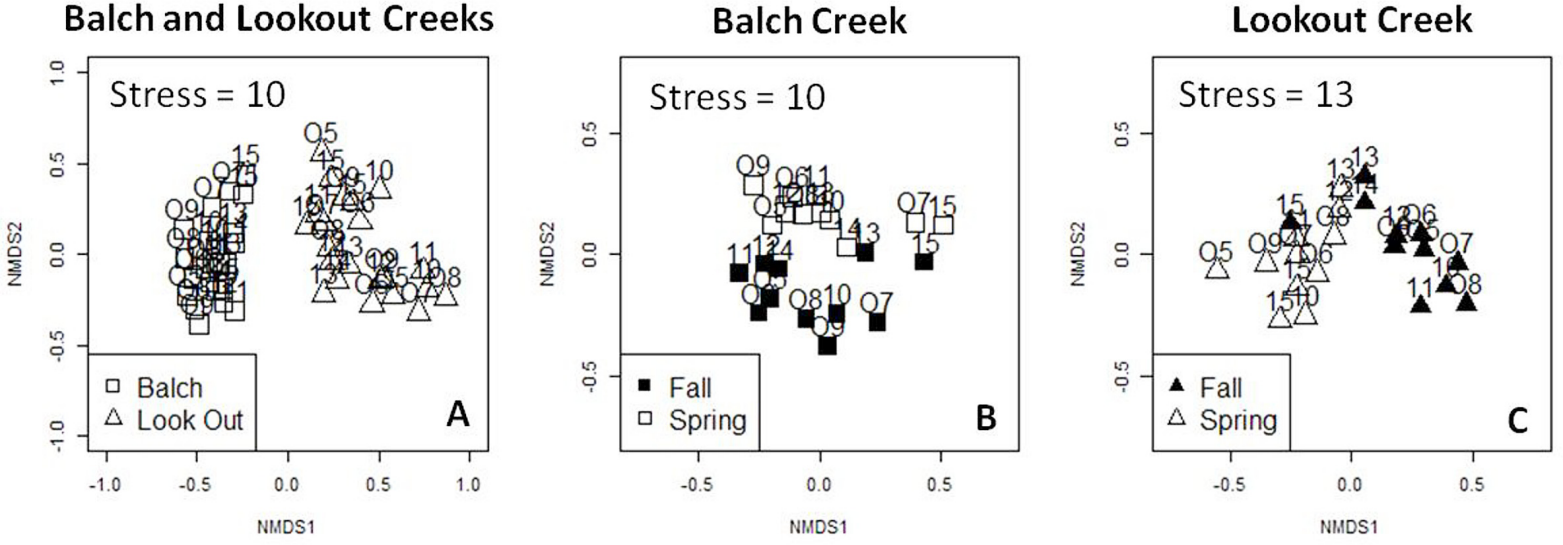
Ordinations for both streams (Panel A), Balch Creek (Panel B) and Lookout Creek (Panel C). Participants generated data for the ordinations by sampling the same reach biannually from 2005–2015. Season is indicated for fall (filled symbol) and spring (open symbol) samples. The two-digit number indicates sampling year.

Ordinations showed significant differences in assemblage between streams and seasons (Fig 6). Invertebrate assemblage was significantly different between streams (ANOSIM, R = 0.88, *P* < 0.01, n = 22 at each stream) and was separated along NMDS axis 1 and varied along NMDS axis 2 (Fig 6A). The ordinations for each stream also showed distinct seasonal patterns. At Balch Creek, seasonal assemblage was significantly different (ANOSIM, R = 0.56, *P* < 0.01, n = 11 for each season) and generally separated along NMDS axis 2 (Fig 6B). Two samples (fall 2013 and fall 2015) were more similar to spring assemblages. At Lookout Creek, seasonal assemblage was also significantly different (ANOSIM, R = 0.67, *P* < 0.01, n = 11 for each season) and separated along NMDS axis 1 (Fig 6C). At Lookout Creek, the fall 2013–2015 samples also appeared to be more similar to spring assemblages.

IBI scores were higher at Lookout Creek and generally stable over the 11-year study period. Median IBI scores were 19 at Balch Creek (range 14–22) and 24 at Lookout Creek (range 20–26). IBI scores were significantly higher at Lookout Creek (Mann Whitney, T = 10, *P* < 0.001, n = 22 at each stream). Seasonal IBI scores were slightly higher in the fall, but this difference was not significant at Balch Creek (Mann Whitney, T = 54, *P* > 0.05, n = 11 for each season) or Lookout Creek (Mann Whitney, T = 57, *P* > 0.05, n = 11 for each season). Two of the six metrics used in the IBI showed very little variation over the study period. In 43 of 44 sampling events at both streams, Percent Diptera and % dominance received a score of 1 and 5 respectively.

### 3.3 Temporal Variability

**Fig 7:**
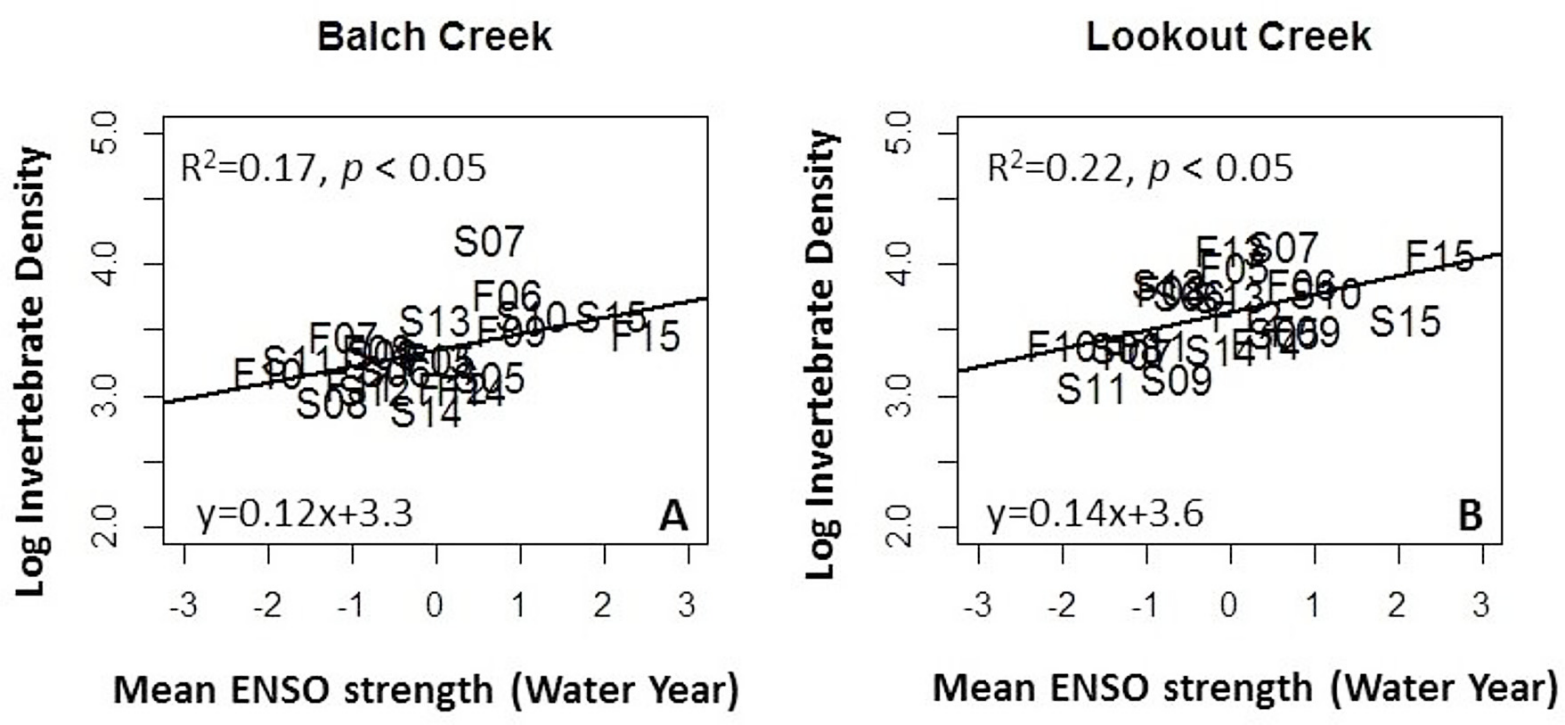
Linear models and R^2^ values of invertebrate density as a function of water-year ENSO MEI at Balch Creek (Panel A) and Lookout Creek (Panel B). Sample points are represented by text with season and year indicated.

From 2005–2015, invertebrate abundance CV was 0.36 at Balch Creek and 0.31 at Lookout Creek. Mean invertebrate abundance between streams was correlated (R = 0.53). Seasonal CV was higher at Balch Creek (0.44 and 0.23 for spring and fall respectively) but not at Lookout Creek. For 20 invertebrate families, CV ranged from 0.27 to 1.7 (Table 3). Linear models (Fig 7) of the invertebrate abundance were positively correlated with water-year ENSO MEI at Balch Creek (Regression, y = 0.12X_ENSO MEI_ + 3.3, R^2^ = 0.17, f = 5.3, *P* < 0.05, d.f. = 20) and Lookout Creek (Regression, y = 0.14X_ENSO MEI_ + 3.6, R^2^ = 0.22, f = 6.8, *P* < 0.05, d.f. = 20). Linear models of invertebrate density as a function of sample-year were not significantly correlated at either creek. The multiple-linear model was significantly correlated at Balch Creek (Regression, y = 0.15X_ENSO MEI_—0.02X_sample-year_ + 3.5, R^2^ = 0.25, f = 4.5, *P* < 0.05, d.f. = 19) and Lookout Creek (Regression, y = 0.15X_ENSO MEI_—0.01X_sample-year_ + 3.8, R^2^ = 0. 21, f = 3.7, *P* < 0.05, d.f. = 19). The LMG model R^2^ was 0.30 at Balch Creek and 0.27 at Lookout Creek with ENSO MEI accounting for the largest percent of explained variance at both streams (Table 4). Using the percent contributions of ENSO MEI and sample-year determined by the LMG model, the long-term CV related to environmental conditions was 0.11 (30%) at Balch Creek and 0.08 (27%) at Lookout Creek (Fig 5).

**Table 3:** CV for common families collected in Balch and Lookout Creeks from 2005–2015 for taxa present in at least 5 samples in each season. Some taxa were only present in either Balch Creek (+) or Lookout Creek (*).

	*Mean Fall Temporal CV*	*Mean Spring Temporal CV*
**Mayfly**		
Ameletidae	0.97*	1.1–1.2
Baetidae	0.59–1.1	0.48–0.98
Ephemerellidae	0.50*	0.59*
Heptageniidae	0.27–0.51	0.32–0.45
Leptophlebiidae	0.76–1.0	0.41–0.96
**Stonefly**		
Chloroperlidae	0.52–0.76	0.58–1.1
Leuctridae	0.98–1.3	NA
Nemouridae	0.48–0.71	0.97–1.1
Perlidae	0.37*	0.63*
Perlodidae	0.40–1.2	0.77–0.93
Pteronarycidae	0.73*	1.1*
Peltoperlidae	0.51*	0.49*
**Caddisfly**		
Glossossomatidae	1.2*	NA
Hydropsychidae	0.66–0.87	0.88–1.7
Limnephilidae	1.4*	0.83–0.86
Rhyacophilidae	0.36–1.0	0.43–0.63
**Diptera**		
Chironomidae	0.86–0.88	0.69–1.4
Simulidae	1.4–1.6	0.78–1.4
**Other**		
Gammaridae	0.94^+^	0.92^+^
Elmidae	0.98–1.1	NA

**Table 4:** Results of the linear model and variance partitioning for the long-term temporal data. R^2^ values are shown for both the linear and LMG regression models. Model parameters (ENSO MEI and sample-year) are expressed as percent explained for the LMG and multiple-linear regression models.

*Creek*	*N*	*Multiple-Linear Model R* ^*2*^	*LMG Model R* ^*2*^	*LMG ENSO MEI*	*LMG Sample-Year*	*LMG Unexplained*
Balch	22	0.25	0.30	22%	8%	70%
Lookout	22	0.21	0.27	25%	2%	73%

## Discussion

### 4.1 Variability of invertebrate data collected by citizens

The purpose of this study was to systematically examine three sources of variability associated with invertebrate data collected by citizen scientists and to consider the value of such data for use in stream management. This was accomplished by characterizing and estimating three sources of variability inherent to citizen invertebrate data and evaluating the use of such data for stream management. At the sample level, the Field method resulted in data that were more variable than the laboratory-based method and biased towards larger invertebrates. This finding is confirmed by other research of non-lethal field methods for collecting invertebrates [4].

Within-reach spatial CV generated by the Field method ranged from 0.43 to 0.50, of which 62% of the variation was due solely to subsampling. Over the 11-year study, temporal CV ranged from 0.31 to 0.36, which is generally lower than CV values reported in other regional studies. For example, in a study of three streams in the Mediterranean climate of California, invertebrate abundance CV ranged from 0.68 to 0.88 [35]. In the present study, temporal variability of invertebrate abundance was partially explained by ENSO MEI strength (R^2^ = 0.17–0.22); these values are slightly higher than the correlation values reported in the California study (r_spearman_ = 0.09–0.16) [35]. The relatively higher CVs and lower correlation with ENSO observed in the California study may be due to its more specific taxonomic focus (e.g. genus vs. family) and multi-habitat sampling design, which would result in more variability and weaker correlations with climate than the riffle-only Field method used in this study. In this study, the ENSO MEI was used as a model parameter because it represents a global climate phenomenon and thus could be used as a worldwide estimate of the climactic contribution to temporally-oriented data generated by citizen programs in other parts of the world. The global application of the findings presented here is supported by the fact that despite the much drier conditions present in the California streams, the correlation of invertebrates with ENSO was similar to wetter and cooler maritime streams of western Oregon. While a more specific analysis of climate condition (e.g. temperature or precipitation) would certainly help connect these findings to regional climactic processes, such analysis is beyond the scope of this study and would not necessarily increase the relevance of the findings for citizen programs in other parts of the world.

### 4.2 The value of citizen data for stream management

The findings of this study support the use of citizen-generated invertebrate data in stream management. The CV observed at each stream was less than 0.5 and for within-reach samples and less than 0.4 for long-term temporal samples. Furthermore, the citizen data was able to detect the influence of environmental conditions that represented relatively small sources of variation (i.e. less than 0.11 CV). These finding support the claim that citizen-generated data would likely detect at least 50% change in mean invertebrate abundance. In addition, some taxa had relatively low CV values (e.g. Heptageniidae, Table 3), thus making them potentially useful as indicator organisms for citizen programs. Such citizen-based indicator taxa should be relatively common, easy to see and identify and have well known ecological information. The correlation of invertebrate abundance with the ENSO MEI and sample-year provides evidence that despite the relatively higher variability, the Field method was still able to detect changes in the invertebrate community associated with broad-scale climactic and environmental conditions. This finding supports the use of citizen data by stream managers to detect changes in stream invertebrates due to climate change or anthropogenic-related stressors. However, the estimates for detection limits presented here only apply if a randomized subsampling technique is used to generate samples with at least 150 invertebrates; otherwise, variability and detection limits will certainly be higher.

Additionally, this study showed that assemblage data generated by the citizen data would also be useful in stream management. This is supported by the consistent patterns observed in the ordinations at each stream and the relative stability of the IBI scores. Ordinations showed that the assemblage was significantly different between streams and for both seasons, which was expected given the different environmental characteristics of the study watersheds. This finding suggests that data generated by citizen groups would detect major differences in assemblage due to watershed or stream condition. Furthermore, this study provides evidence that assemblage could also be used to monitor the effect of climate change on stream ecosystems. This can be seen in the ordinations at both streams where three fall samples (2013–2015) were more similar to spring samples. This shift towards a spring assemblage may be explained by the relatively high ENSO MEI scores associated with these samples. Indeed, the 2013–2015 fall samples represent 3 of the 7 positive ENSO MEI values observed in this study.

IBI scores were relatively stable over the 11-year study period. This would be expected in the absence of any major disturbance in the study streams or watersheds, which did not occur during the study period. At Balch Creek, 86% of IBI scores fell within the moderately disturbed category, which was expected given the moderate land development and urban location. At Lookout Creek, because the IBI scores were near the threshold score of the unimpaired category, only 60% of the IBI scores were within the expected unimpaired category; however, 95% of the IBI scores at Lookout Creek were within one point (e.g. 22) of the unimpaired category. Two of the six metrics used in calculating IBI scores appear to be problematic when generated with citizen data. Both % Diptera and % Dominance showed little variation at either stream. This is likely due to the challenge of seeing small organisms in the field, which results in downward bias in total richness and the scores associated with the % Diptera and % dominance metrics. Given this bias, citizen data would be more likely to underestimate, rather than overestimate, stream condition when using similarly constructed biotic metrics. The observed bias in IBI scores could be addressed by recalibrating metrics to reflect the limitations of sorting and identifying small invertebrates in the field.

The overall findings of this study support the use of citizen-generated invertebrate data for non-regulatory purposes in stream management. For example, citizen data could be used to monitor temporal or spatial changes in the invertebrate community, document the impact of environmental restoration efforts or provide an early-warning signal that professional monitoring should be implemented. The findings of this study are also relevant to other citizen-based programs that may use a slightly different nonlethal technique (i.e. larger substrate area or different subsampling methods). For example, programs that use a rare and large species-screening protocol to search for important taxon that may not be part of a subsample. In this case, the Field method can be easily modified to include this extra step.

### 4.3 Use of the Field method for citizen-based science programs

From a citizen science perspective, there are several advantages to using a field-based invertebrate collection method. First, the Field method reflects several elements known to be important for long-term success of citizen science programs including: methods that are specifically designed for the non-professional setting, data that are accurate and meaningful and findings that are applied to stream management efforts [6, 39, 40]. Another benefit of the Field method is its cost and flexibility. Using simple and inexpensive equipment, the Field method can accommodate varying numbers of participants or minimum count requirements. For example, large groups can work together to collect multiple samples or smaller groups can continue to sample until a minimum count is reached. Furthermore, the nonlethal approach of the Field method allows participants to examine live insects and return them to the stream unharmed, thereby promoting stewardship and modeling an environmental ethic.

There are several limitations of this study and its findings that are worth considering. All investigations were conducted in generally small, high-gradient streams with rocky substrates, clear cold water and relatively little organic debris. Using the Field method in streams with slow flow, silty substrate, turbid water or abundant organic matter may result in higher variability and thus increase detection limits. Furthermore, the temporal correlation of climate with invertebrates should be interpreted with caution because the relationship may be due to other environmental factors, such as body size and phenology [41], which make the invertebrates more likely to be captured in D-nets or visible to the naked eye. Several strategies can be used to reduce the potential variability and increase the reliability of data generated by citizen groups using the Field method. The most important is to make sure that the methods are used consistently; modifications to the protocol can drastically affect the reliability of the data. Another important consideration is related to invertebrate identification and counting in the field. Because the invertebrates are returned to the stream, it is not possible to verify invertebrate identifications at a later date. Therefore, a person trained in family-level taxonomy should always be present to verify counts and identifications before samples are returned to the stream. In this regard, the technique presented here represents more of a blended citizen science approach wherein citizens work side-by-side with a trained taxonomist to generate datasets. In either case, voucher specimens can be used to correctly identify specimens at a later date. Furthermore, the participation of a stream manager or experienced scientist on data collection field trips would also serve to help build connections between citizens and the scientific community.

Citizen science programs have the potential to supply large amounts of useful invertebrate data to stream managers, particularly in streams for which no other data exists. This study presents important information about the potential variation in citizen data and presents estimates for the detection limits of such data. The findings of this study suggest that citizen-based biomonitoring programs can provide useful information to stream managers about changes in stream invertebrate communities over time and across stream sites. It is hoped that the main benefit of this research will be to encourage partnerships between stream managers and citizen groups to improve stream quality and promote stewardship and public engagement in environmental issues.

## Supporting Information

S1 Fig Procrustes residuals. Circles are Lab samples with the arrow pointed to corresponding Field samples.
(TIF)

S1 File Experimental data. (CSV)
