# Correction: The Value of Long-Term Stream Invertebrate Data Collected by Citizen Scientists

**DOI:** 10.1371/journal.pone.0156435

**Published:** 2016-05-23

**Authors:** The PLOS ONE Staff

## Notice of Republication

This article was republished on May 13, 2016, to correct errors that were introduced during preparation of the manuscript for publication: Figs 5–7 were swapped and S1 Fig was incorrect. The publisher apologizes for the errors. Please download this article again to view the correct version. The originally published, uncorrected article and the republished, corrected article are provided here for reference.

## Supporting Information

S1 File Originally published, uncorrected article. (PDF)

S2 File Republished, corrected article. (PDF)
