# Supplementary figures and images for: The Value of Long-Term Stream Invertebrate Data Collected by Citizen Scientists
Source: PLoS One. 2016 Apr 27;11(4):e0153713. doi: 10.1371/journal.pone.0153713 (PMC4847766; doi:10.1371/journal.pone.0153713)

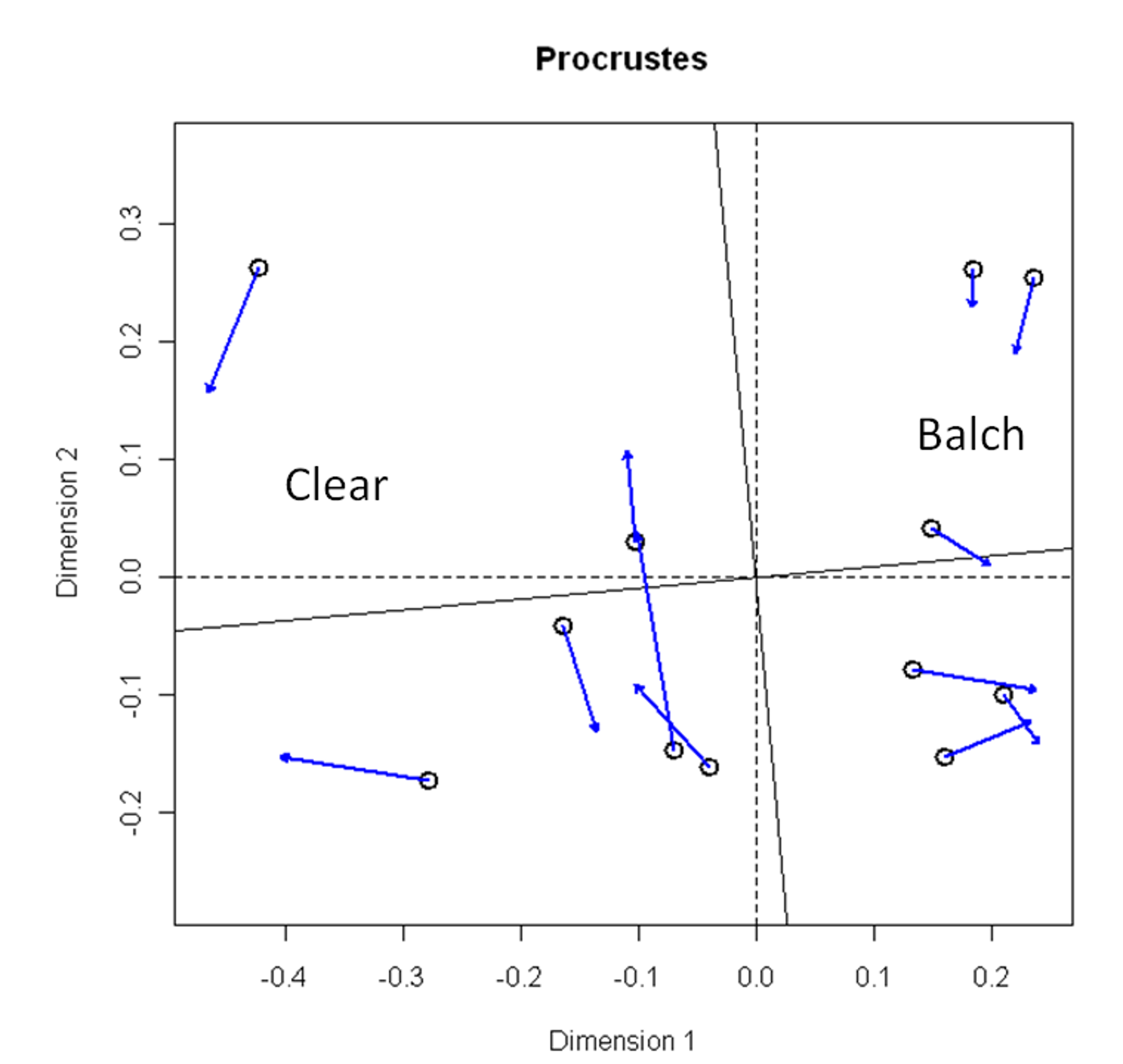

Supplement: S1 Fig — Circles are Lab samples with the arrow pointed to corresponding Field samples. (TIF) [file pone.0153713.s001.tif]
